# Biomarkers of Metabolism in Amyotrophic Lateral Sclerosis

**DOI:** 10.3389/fneur.2019.00191

**Published:** 2019-03-18

**Authors:** Siobhan E. Kirk, Timothy J. Tracey, Frederik J. Steyn, Shyuan T. Ngo

**Affiliations:** ^1^The Australian Institute for Bioengineering and Nanotechnology, The University of Queensland, Brisbane, QLD, Australia; ^2^Centre for Clinical Research, The University of Queensland, Brisbane, QLD, Australia; ^3^Queensland Brain Institute, The University of Queensland, Brisbane, QLD, Australia

**Keywords:** amyotrophic lateral sclerosis, ALS, metabolism, biomarker, motor neurone disease

## Abstract

Amyotrophic lateral sclerosis (ALS) is a neurodegenerative disorder characterized by the deterioration of motor neurons. However, this complex disease extends beyond the boundaries of the central nervous system, with metabolic alterations being observed at the systemic and cellular level. While the number of studies that assess the role and impact of metabolic perturbations in ALS is rapidly increasing, the use of metabolism biomarkers in ALS remains largely underinvestigated. In this review, we discuss current and potential metabolism biomarkers in the context of ALS. Of those for which data does exist, there is limited insight provided by individual markers, with specificity for disease, and lack of reproducibility and efficacy in informing prognosis being the largest drawbacks. However, given the array of metabolic markers available, the potential exists for a panel of metabolism biomarkers, which may complement other current biomarkers (including neurophysiology, imaging, as well as CSF, blood and urine markers) to overturn these limitations and give rise to new diagnostic and prognostic indicators.

## Overview

Amyotrophic lateral sclerosis (ALS) is a progressive neurodegenerative disease caused by the death of motor neurons in the brain and spinal cord. The loss of neuronal input leads to progressive paralysis and patient mortality within 2–5 years from diagnosis (1). ALS likely arises from a combination of genetic susceptibility and environmental exposures (2, 3), although it is recognized that ALS is a complex, multi-system disease (4, 5).

Given the complex and heterogeneous nature of ALS, diagnosis and tracking of prognosis remains difficult. Current diagnostic criteria typically follow tests to rule out other pathological causes of symptoms and include: indicators of upper and lower motor neuron involvement, nerve conduction tests, electromyography and “watchful waiting” (4). As a result, researchers have attempted to utilize a wide range of biomarkers—observable biological measurements that confirm the presence or progression of a change in body status, as a means of diagnosing and following disease progression. While the current range of biomarkers in ALS offer some diagnostic and prognostic benefit, there is a need to identify a biomarker that satisfies the following six attributes: specificity to disease; reproducibility; appearance early in the disease; stability across the diurnal period; independence of dietary status and behavior; and a notable change during disease progression. By meeting these criteria, a biomarker can be used to reliably identify and track disease progression, in a manner that can easily be reproduced in a clinical setting.

**Figure 1:**
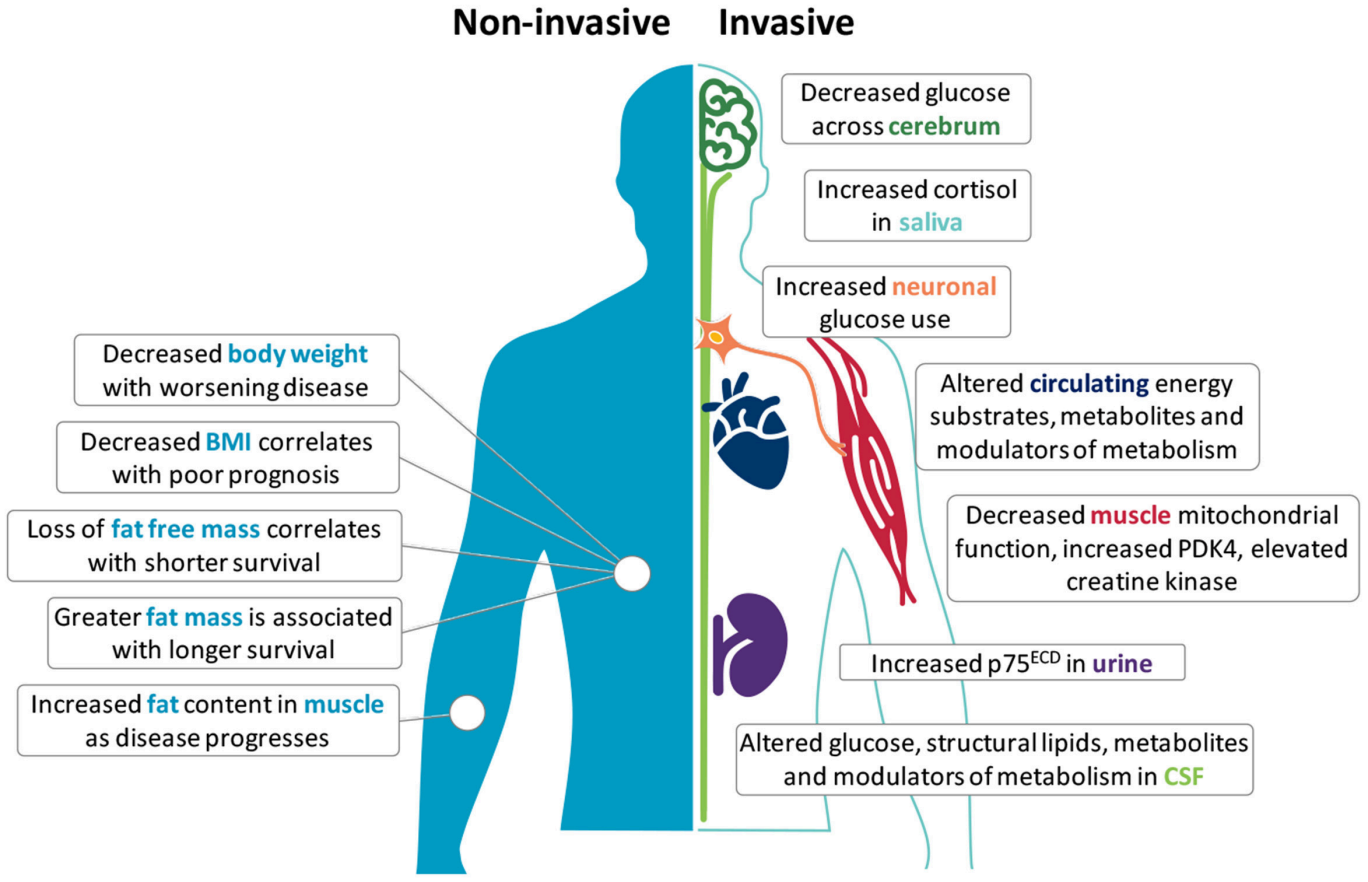
Potential metabolism biomarkers in amyotrophic lateral sclerosis (ALS). Metabolic alterations in ALS offer opportunities to use metabolism biomarkers for the diagnosis, categorization, and tracking of disease. Non-invasive anthropometric measures include body weight, body mass index (BMI), fat free mass, fat mass, and fat distribution. Invasive measures include the use of F18-PET to assess glucose metabolism in the central nervous system, or require the sampling of saliva, blood, cerebrospinal fluid (CSF), muscle tissue, and urine. Although few independent markers are specific, reproducible or able to track disease in ALS, used together with complementary biomarkers (including neurophysiology and imaging), these markers may provide deeper insights into metabolic perturbations that are potentially involved in the onset and progression of disease.

Metabolic perturbations occur in ALS patients and in mouse models of the disease; both at the systemic and cellular level (6, 7). Clinically, an increase in resting energy expenditure (REE) and decline in body mass index (BMI) is linked to worse outcome (8–10), suggesting prognostic potential in metabolic biomarkers. Given that changes in metabolic status are generally reflected in overall body weight, body composition, and tissue/cellular metabolic function, metabolic changes at the anthropometric, tissue and cellular levels may represent appreciable metabolism biomarkers of ALS onset, progression, and/or severity (Figure 1). A list of the potential biomarkers of metabolism in ALS, and their quality relative to the aforementioned identifying attributes are summarized in Table 1.

**Table 1:** Classification of potential biomarkers of metabolism in amyotrophic lateral sclerosis (ALS).

**Marker**	**Observation**	**Utility as a biomarker in ALS**						**Biomarker Score**	**References**
		**Specific to ALS**	**Reproducible**	**Pre-diagnostic**	**Diurnal stability**	**Independence**	**Change with progression**		
**ANTHROPOMETRIC MARKERS**									
Body mass index (BMI)	Lower BMI is an indicator of poor prognosis. U-shaped association; lower BMI is associated with increased risk and faster progression whereas BMI in the range of morbid obesity is associated with shorter survival. Degree of premorbid loss of BMI predicts risk of ALS	N	N	Y	Y	N	*Variable*	**2.5**	(8, 11–22)
Body weight	Weight loss correlates with faster disease progression; weight loss suggested as a risk factor for ALS	N	N	N	Y	N	*Variable*	**1.5**	(14, 21, 23–27)
Fat mass	Fat mass at diagnosis is not a determinant of survival. Increased fat mass is correlated with longer survival	N	N	*Insufficient data*	Y	N	Y	**2**	(14, 28)
Fat free mass	Fat free mass at diagnosis is not a determinant of survival. Loss of fat free mass is associated with shorter survival	N	Y	*Insufficient data*	Y	N	Y	**3**	(14, 23)
Fat distribution	Redistribution and increased deposition of fat in muscle	N	*Insufficient data*	*Insufficient data*	Y	N	*Insufficient data*	**1**	(29)
**IMAGING MARKERS**									
Brain glucose use	Hypometabolism specific to select brain regions; varies between studies	N	*Insufficient data*	*Insufficient data*	*Likely*	*Likely*	Y	**2**	(30–33)
Spinal cord glucose use	Hypermetabolism; changes in glucose metabolism correlates with disease progression	N	*Insufficient data*	*Insufficient data*	*Likely*	*Likely*	*Variable*	**1.5**	(34, 35)
**MUSCLE MARKERS**									
Creatine kinase	Increased in blood; variability in correlation with disease progression/survival. Greater increase observed in male subjects and limb-onset ALS	N	Y	Y	*Likely*	*Likely*	*Variable*	**3.5**	(36–43)
Mitochondrial function	Decreased activity of complex I and IV. Activity also declines over course of disease	N	*Insufficient data*	*Insufficient data*	Y	Y	*Variable*	**2.5**	(44, 45)
PDK4 levels	Increase in pyruvate dehydrogenase kinase 4 (PDK4) correlated with increased denervation and fuel switch	N	*Insufficient data*	*Insufficient data*	Y	Y	*Likely*	**2.5**	(46)
Glucose	Increased	N	N	*Insufficient data*	N	N	N	**0**	(47)
Sphingolipids	Increased	N	*Insufficient data*	*Insufficient data*	N	N	Y	**1**	(48)
Phosphatidylcholine	Increased	N	*Insufficient data*	*Insufficient data*	N	N	N	**0**	(48)
Cholesterol + Carriers	Increased	N	*Insufficient data*	*Insufficient data*	N	N	N	**0**	(49)
Lactate	Increased	N	*Insufficient data*	*Insufficient data*	N	N	*Insufficient data*	**0**	(47, 50)
**CEREBROSPINAL FLUID (CSF) MARKERS**									
Pyruvate	Increased	N	*Insufficient data*	*Insufficient data*	N	N	*Insufficient data*	**0**	(51)
Insulin	Decreased	N	*Insufficient data*	*Insufficient data*	N	N	N	**0**	(52)
Growth hormone	Decreased	N	*Insufficient data*	*Insufficient data*	N	N	N	**0**	(52)
**CIRCULATING MARKERS (BLOOD, PLASMA AND SERUM)**									
Glucose	Increased (33% of patients achieve World Health Organization (WHO) criteria for impaired glucose tolerance)	N	N	*Insufficient data*	N	N	N	**0**	(53)
Mannose	Increased	N	N	*Insufficient data*	N	N	*Insufficient data*	**0**	(54)
Free fatty acids	Increased	N	N	*Insufficient data*	N	N	N	**0**	(53)
Sphingolipids	Increased	N	N	*Insufficient data*	N	N	N	**0**	(54)
Cholesterol + Carriers	Major variations and contradictory reports mask any specific trend	N	N	*Insufficient data*	N	N	*Variable*	**0.5**	(53, 55–62)
β-hydroxy-butyrate	Increased	N	N	*Insufficient data*	N	N	*Insufficient data*	**0**	(63)
2-hydroxy-butyrate	Increased	N	N	*Insufficient data*	N	N	*Insufficient data*	**0**	(54)
α-ketoglutarate	Increased	N	N	*Insufficient data*	N	N	*Insufficient data*	**0**	(54)
Acetate	Increased	N	N	*Insufficient data*	N	N	*Insufficient data*	**0**	(63)
Adiponectin	Increased	N	N	*Insufficient data*	N	N	N	**0**	(64)
Cortisol	Increased	N	N	*Insufficient data*	N	N	N	**0**	(65)
Cortisol (morning peak)	Decreased	N	N	*Insufficient data*	N	N	N	**0**	(65)
Insulin	Decreased	N	N	*Insufficient data*	N	N	N	**0**	(52, 64)
Gastric inhibitory peptide	Decreased	N	N	*Insufficient data*	N	N	N	**0**	(64)
Ghrelin	Decreased	N	N	*Insufficient data*	N	N	N	**0**	(64, 66)
**SALIVA MARKERS**									
Cortisol (night-time)	Increased	N	*Insufficient data*	*Insufficient data*	N	*Likely*	*Insufficient data*	**0.5**	(67)
Cortisol (Stress-induced)	Decreased	N	*Insufficient data*	*Insufficient data*	N	*Likely*	*Insufficient data*	**0.5**	(67)
Cortisol (circadian rhythm)	Decreased	N	*Insufficient data*	*Insufficient data*	N	*Likely*	*Insufficient data*	**0.5**	(67)
**URINE MARKERS**									
p75 neurotrophin receptor extracellular domain	Increased	N	*Likely*	*Insufficient data*	Y	Y	Y	**3.5**	(68)

*The strength of proposed biomarkers are scored relative to their potential to serve as markers that are specific to ALS, and that conform to the requirements as detailed in text*.

*Specific to ALS refers to uniqueness of the marker to ALS over other diseases, reproducible refers to whether the indicated change is reproducible across patient cohorts, pre-diagnostic indicates where changes are apparent prior to symptom onset, diurnal stability refers to the consistency of the marker throughout the day, independence indicates the ability of the marker to remain stable regardless of changes in food intake or behavior, change with progression identifies whether the marker changes as disease progresses. For each potential biomarker, a score out of 6 was determined (biomarker score, indicated in bold), where Y (Yes) = 1 point, N (No) = 0 points, Variable = 0.5 points, Likely (supported by animal or statistical modeling studies) = 0.5 points, and Insufficient data = 0 points*.

## Anthropometric Body Measures

Lower premorbid BMI is associated with increased risk for ALS (11–13), and the degree of decline in premorbid BMI predicts ALS risk and survival (14, 15). Lower BMI, or a decline in BMI following diagnosis correlates with worse survival (16, 17), although this association is not always observed (18, 19, 23, 24). Rather, the mortality risk for ALS relative to BMI exists as a U-shaped curve, in which mortality decreases with increasing BMI, until BMI levels indicate premorbid obesity. Thereafter, mortality risk increases again (8, 20). This seemingly complex association could be explained by changes in body composition throughout disease progression.

BMI is often used as an indirect measure of fatness. However, conventional anthropometric measures of BMI and body adiposity index (BAI) do not always accurately reflect changes in fat and/or fat free mass (FFM) in ALS (69). In this regard, fat mass (FM) and FFM at diagnosis are not associated with survival risk (14), yet redistribution of adipose tissue does occur in ALS (29), and visceral fat is correlated with functional status and survival (28). Moreover, serial assessment of body FM indicates that increases in FM are associated with longer survival (14). While a decrease in FFM serves as an independent prognostic factor for shorter survival in ALS (23), we did not identify any studies that document progressive changes in muscle mass as a potential marker of disease progression in ALS. As a hallmark of ALS, however, there is potential to use the loss of FFM as a marker of disease progression. Such measures must consider the technical difficulties associated with assessing FFM in patients who experience significant and progressive disability, while also accounting for whole body and regional changes in FFM, which differ greatly between patients.

Despite BMI and BAI being poor predictors of body composition in ALS, changes in BMI may offer reliable measures for progressive changes in the overall nutritional status of the patient, and by proxy, disease progression. As documented by Kasarskis et al. a progressive decline in body weight is commonly observed in ALS patients in the months prior to death, and this reduction in body weight or BMI likely reflects a state of undernutrition (25). In recent years, lower BMI has been found to be associated with lower ALSFRS-R scores (70), and a loss of body weight (14, 21, 23, 24, 26, 27, 71) and BMI (14, 17, 22, 24) throughout disease course is consistently associated with shorter survival. Not surprisingly, these observations, while serving as markers for disease progression, have resulted in the adoption of interventions aimed at slowing weight loss in ALS (72).

## Skeletal Muscle Pathology

With findings suggesting that FFM is a prognostic factor in ALS (23), analysis of skeletal muscle, the primary component of FFM, may offer insights into tissue-specific metabolism biomarkers. Assessment of cellular metabolic changes in skeletal muscle can be challenging, especially when weighing the clinical benefit against that of an invasive procedure on a patient undergoing significant muscle wasting. Furthermore, heterogeneity in site of disease onset leads to variable muscle pathophysiology between patients (73).

Despite these limitations, creatine kinase, an enzyme that is linked with muscle damage and deterioration, has been studied intensely in ALS. While not strictly a metabolic marker, creatine kinase can be considered as an important modulator of body composition (74). As such, it may indirectly influence systemic metabolic processes. Numerous reports of increased creatine kinase in ALS (36–43), and particularly in limb-onset patients (38, 43), highlight the potential for its use as a marker of disease. However, contradictory observations of associations between creatine kinase and clinical parameters of disease, and disease progression and survival attest to the need for further investigations into determining the utility of creatine kinase as a biomarker in ALS.

## Mitochondrial Dysfunction

In human ALS muscle, mitochondrial defects including dysregulation of respiratory complex I (44), decreased respiratory complex I and IV activity (45, 75), decreased muscle mitochondrial protein expression (75) and upregulation of muscular mitochondrial uncoupling protein 3 (76) indicate that impairments in mitochondrial function could serve as a metabolic marker of ALS. It should be noted, however, that these studies were unable to correlate mitochondrial defects with functional parameters of disease progression, despite studies in animal models reporting a strong relationship between the two (77–79). Therefore, while there is clear evidence of mitochondrial defects in ALS, mitochondrial defects *per se* cannot currently be used as a biomarker due to the difficulty in both easily observing these defects in a clinical setting, and linking such defects to a marker of disease progression and/or survival. Instead, emphasis could be placed on the assessment of the more easily detectable metabolites that drive mitochondrial function.

## Glucose Metabolism

Glucose use in the brain of ALS patients has been evaluated using fluorodeoxyglucose F18 positron emission tomography (F18-PET) (30–33). These studies have identified decreased glucose use in the primary motor cortex of ALS patients, suggesting that this brain region is hypometabolic (32). Other studies have reported a decrease in the use of glucose across other brain regions (31, 33); although this may reflect the differences in experimental cohorts. In this regard, Claassen et al. investigated a cohort of patients with primary lateral sclerosis, while the study by Ludolph et al. evaluated ALS patients with both upper and lower motor symptoms. Given that the degree of cerebral hypometabolism in ALS is correlated with the duration of clinically-identified symptoms (30), the ability of the motor cortex to utilize glucose may allow for monitoring of disease progression. However, since brain glucose hypometabolism is not specific to ALS (80), its use as a diagnostic/prognostic marker is limited.

F18-PET has also been used to assess the uptake and utilization of glucose in the cervical spinal cords of ALS patients (34, 35, 81). Overall, observations of spinal cord glucose hypermetabolism (34, 35, 81) is congruent with increased levels of glucose in the CSF of ALS patients (47). In a study by Yamashita et al. glucose hypermetabolism on the ipsilateral side to the patient's symptoms was found to be positively correlated with ALSFRS-R, suggesting that changes in spinal cord glucose metabolism are specific to the affected corticospinal tract and the degree of disease severity (35). By contrast, the study by Marini et al. reported spinal cord glucose hypermetabolism independent of disease duration and functional impairment (34). As such, the degree of glucose use in the spinal cord may present some use for diagnostic testing, but provides limited insights for evaluation of disease progression and prognosis. Indeed, glucose hypermetabolism in the spinal cord extends to other neurological conditions (82, 83), thereby limiting its use as a specific biomarker for ALS. Finally, as the reproducibility of F18-PET in both the brain and spinal cord is low (84), more rigorous testing is required to determine if results are consistent across a heterogeneous ALS population.

Alterations in glucose metabolism in ALS extend beyond the central nervous system (CNS). Glucose tolerance tests conducted by Pradat et al. indicate that ALS patients have a significant increase in blood glucose levels following the provision of a glucose load when compared to age- and sex-matched controls. Within ALS patients, a degree of heterogeneity was observed, with 33% of participants meeting World Health Organization criteria for impaired glucose tolerance (53). Impaired glucose tolerance is in line with reports of insulin resistance in ALS (85), and could explain observations of increased expression of pyruvate dehydrogenase kinase 4 (PDK4) in skeletal muscle of ALS patients (46). Similarly, mannose, an epimer of glucose that has recently been shown to be a predictor of insulin resistance (86), has been reported to be significantly increased in the plasma of ALS patients (54). While the assessment of glucose tolerance and insulin resistance is relatively straightforward, these tests lack reproducibility and specificity to ALS (87–89). Therefore, although glucose metabolism is altered in ALS, it cannot be used as an independent biomarker for ALS diagnosis and prognosis.

## Fatty Acids and Ketones

In patients with ALS, the resting level of circulating free fatty acids (FFAs) is significantly increased (53). While higher levels of FFAs has been linked to impaired glucose tolerance in ALS, it has not been shown to be correlated with any markers of disease progression or severity. Ketones, including β-hydroxy-butyrate (63) and 2-hydroxy-butyrate and α-ketoglutarate (54), which are produced through fatty acid metabolism under fasting conditions, are also significantly increased in ALS. Similar to FFAs, no correlations have been observed between disease status and the expression of ketones. Thus, FFAs and ketones cannot currently be considered as reliable biomarkers for ALS, and the lack of specificity for ALS-centric pathology indicate that they may not present as particularly valuable diagnostic markers individually.

## Downstream Metabolites

Metabolites, the downstream indicators of metabolic function, are also impacted in ALS. While not specific to ALS, altered expression of metabolites may offer a potential avenue for biomarker discovery. In line with disease heterogeneity, reported levels of metabolites in the blood and CSF are variable. Notably, the levels of lactate (47, 50) and pyruvate (51) in the CNS are increased, potentially reflecting an increase in metabolic output, or increased release of metabolites into the CSF following neuronal deterioration. Given that mitochondrial dysfunction is observed in ALS, further evaluation of the ratio between these metabolites may hold significant informative value in ALS due to the diagnostic value of this test for mitochondrial disorders (90).

Blood levels of acetate are increased in ALS (63), although this is not readily observed in the CSF (47, 51). Acetate is a key metabolite in the oxidation of fatty acids. As acetate synthesis precedes the formation of citric acid in the Krebs cycle, changes in circulating acetate may occur due to excess production via an increase in fatty acid oxidation, increased release from deteriorating muscle cells, or other disruptions to mitochondrial membrane integrity (e.g., due to the presence of free radicals). Such potential mechanisms align with ALS pathology. As a whole, downstream metabolites hold promise as potential biomarkers, and further work that can interrogate relationships between metabolites and clinical parameters of disease would add merit to their use as metabolic biomarkers of disease.

## Endocrine Modulators of Metabolism

Insulin is an anabolic hormone that has been reported to be decreased in the blood (64) and CSF (52) of ALS patients. By contrast, other studies have reported no significant differences in plasma insulin levels in ALS patients (91, 92). Other anabolic hormones that have been found to be decreased in ALS include growth hormone (in CSF and blood) (52, 92–94) and gastric inhibitory peptide in blood (64). Conversely, hormones that promote catabolism, such as cortisol (65, 67), and adiponectin (64) are increased or dysregulated in saliva and blood of patients with ALS. Furthermore, ghrelin, an important modulator of appetite, is also reduced in the plasma/blood of ALS patients (64, 66). Given that alterations in these hormones are likely to be symbolic of a change in metabolic function/homeostasis, studies that confirm a link between endocrine markers of metabolism and clinical markers of disease offer potential for their development as prognostic biomarkers.

## Metabolism of Structural Lipids

While fatty acids and their derivatives serve as energy substrates through mitochondrial respiration, they also play an essential role in maintaining cellular integrity. Phospholipids, particularly phosphatidylcholine, are significantly increased in the CSF of ALS patients (48). Sphingolipids, such as stearoyl sphingomyelin and ceramide, are also increased in patient blood (48, 54). Interestingly, in the study by Blasco et al. predictions of clinical measurements, such as ALSFRS-R, were found to be correlated to CSF sphingomyelins and triglycerides with long-chain fatty acids (48). Such findings are favorable for the development of biomarker assays, but further tests are required to confirm the reliability of predictive models, before use as a prognostic biomarker.

An increase in cholesterol esters has been observed in ALS patient spinal cord (95). However, cholesterol and its carriers prove to be more difficult to characterize, with variable levels of HDL and LDL cholesterol being reported in ALS. In a population-based longitudinal study, a positive association was found between LDL cholesterol and ALS risk (55), however, there was no indication of the impact of LDL on disease progression or mortality. Nonetheless, this could serve as a diagnostic biomarker for ALS risk. Previously, higher levels of cholesterol, LDL, as well as an elevated LDL/HDL ratio in ALS patient blood have been correlated with increased survival (56–58). Conversely, similar increases in total cholesterol, LDL, and HDL cholesterol in ALS patient blood (59, 60) and CSF (49) have not been found to be correlated with disease progression. Furthermore, a small number of studies contradict these findings, reporting that cholesterol, LDL, and HDL levels do not vary between ALS patients and controls (53, 61, 62), although lower levels of serum lipids may correlate with worse respiratory function (61). Based on these contradictory observations, the validity of cholesterol as a biomarker remains uncertain. Further studies that address these disparate data are required.

## Novel Metabolism-associated Biomarkers

p75 neurotrophin receptor (NTR) belongs to the tumor necrosis factor family of receptors. It is a transmembrane receptor which binds neurotrophins and pro-neurotrophins (96). p75NTR has been implicated in processes of energy expenditure (97), glucose uptake, and insulin sensitivity (98). In ALS, the secretion of the extracellular domain of p75NTR (p75^ECD^) in urine was recently established as a biomarker for disease progression and prognosis (68, 99). Urinary p75^ECD^ increases as disease progresses, and an elevation of urinary p75^ECD^ is observed alongside a decrease in ALSFRS-R scores (68). While it is not clear if increases in urinary p75^ECD^ in ALS match metabolic derangements that accompany disease progression (such as changes in energy metabolism, glucose uptake and insulin sensitivity), the introduction of p75^ECD^ as a fluid biomarker in ALS provides an opportunity for the evaluation and possible co-development of metabolism-associated biomarkers.

## Conclusion

The complexity and heterogeneity of disease between patients limits the scope for the use of a single reliable biomarker of ALS. Significant changes in metabolism seen in ALS may represent a potential avenue for biomarker development. As documented in this review, a range of markers might be relevant (Figure 1). However, as investigations into the cause for metabolic derangements in ALS are ongoing, and little emphasis has been placed on the development of metabolism biomarkers as diagnostic or prognostic indicators, few reliable metabolism biomarkers exist (Table 1). Moreover, because metabolic alterations in ALS likely arise from the dysregulation of a number of processes, the utility of biomarkers for assessing early or progressive changes in the metabolic state of ALS patients would necessitate the development of a panel that captures the spectrum of metabolic changes that occur at the systemic and cellular level.

As there is no single biomarker for ALS that sufficiently meets the six major attributes of a biomarker, it is clear that the assessment of biomarkers that cover multiple dimensions of the disease is needed in order to generate a comprehensive view of the state of disease. The complementary assessment of metabolism markers alongside other biomarkers including neurophysiology, imaging, as well as CSF, blood, and urine markers may form a more convincing and reliable diagnostic/prognostic platform, while providing insights into the multifactorial nature of disease.

## Author Contributions

SEK, TJT, FJS, and STN conducted the literature search and wrote the manuscript. FJS produced all artwork. STN critically revised the manuscript.

### Conflict of Interest Statement

The authors declare that the research was conducted in the absence of any commercial or financial relationships that could be construed as a potential conflict of interest.
